# Cloning and expression analysis of the DEAD-box/RNA helicase *Oslaf-1* in *Ovomermis sinensis*

**DOI:** 10.1371/journal.pone.0192101

**Published:** 2018-02-06

**Authors:** Siying Tao, Zhenlong Jiao, Guigui Wen, Lihong Zhang, Guoxiu Wang

**Affiliations:** Hubei Key Laboratory of Genetic Regulation and Integrative Biology, School of Life Sciences, Central China Normal University, Wuhan, China; Shanghai Ocean University, CHINA

## Abstract

*Ovomermis sinensis* is a potentially-valuable nematode for controlling insect pests. The parasitic stage of the nematode absorbs nutrients in its host’s hemolymph to maintain its growth development and then kills the host when it emerges. At present, little known about its reproductive development, particularly the responsible molecular mechanism. More detailed research on the genes of reproductive development will not only help us understand the mechanisms underlying sex differentiation in the nematode, but would also be valuable for successfully cultivating them in vitro and using them for biocontrol. In this study, we used the homology cloning method to clone the full-length cDNA of a DEAD-box family gene (*Oslaf-1*) from *O*. *sinensis*. Then, using qRT-PCR technology to detect the expression pattern of the *Oslaf-1* gene at different development stages and tissues, the gene was found to be highly expressed in the post-parasitic stage (P < 0.01) and ovarian (P < 0.05) of *O*. *sinensis*. Western blot analysis showed the same result that the gene is associated with gonadal development and function, but is not gonad-specific. In situ hybridization further demonstrated that the gene is widely expressed in early embryos and is mainly distributed in the gonadal area. However, the signal was mainly concentrated in the reproductive primordia in pre-parasitic juveniles. RNA interference (RNAi) studies revealed that the sex ratio of *O*. *sinensis* soaked in dsRNA of *Oslaf-1* was not statistically different than the *gfp* dsRNA treated groups. Our results suggest that *Oslaf-1* may play a vital role in the reproductive systems of the nematode. In addition, we speculate that the *Oslaf-1* gene plays an important role during embryonic development and that it occurs and develops in the gonads of pre-parasitic juveniles of *O*. *sinensis*.

## Introduction

*Ovomermis sinensis* is a mermithid nematode that parasitizes a broad range of lepidopteran pests, including *Helicoverpa armigera* [1]. Pre-parasitic juveniles of mermithids search for and enter their insect hosts, then develop in the hemocoel of hosts. The fully developed juveniles (parasites juveniles) emerge through the host’s integument, kills the host, and then enters the soil to develop from post-parasite juveniles into adults. Male and female adults mate and lay eggs to complete their life cycle [2]. Because the parasitism rate of mermithid nemotodes (and *O*. *sinensis* in particular) is equal to the host’s mortality rate, they have considerable potential for biologically controlling insect pests [2, 3].

*O*. *sinensis* has a strong ability to adapt to its environment. Sex differentiation in *O*. *sinensis* is determined by environmental cues (called environmental sex determination or ESD), in contrast to genotypic sex determination (GSD), which is the more common mechanism in animals. In controlled experiments, *O*. *sinensis* became females when the parasitic intensity was less than 10, while they developed into males when it was more than 40. In other words, parasitic intensity (or its nutritional requirements in the parasitic stage) is an important factor determining sex differentiation of *O*. *sinensis* [4, 5]. At present, most research has focused on macro-morphology and molecular mechanisms. Because there has been no research focused on how to induce a large number of nematodes to control pests, we intended to find a method to successfully culture *O*. *sinensis* in vitro. Our research also advances the science of sex differentiation and its relationship to functional genes.

DEAD-box family proteins are a putative, ATP-dependent, RNA helicase that involves various stages of RNA processing and RNP remodeling. DEAD-box proteins are defined by their conservation motifs, including the D–E–A–D (Asp–Glu–Ala–Asp) [6]. Vasa, PL10, P68, and eIF4A sub-families are members of DEAD-box family of proteins. *C*. *elegans* is a well-known model organism for this protein family. The *laf-1* gene of *C*. *elegans* is a negative regulator of the *tra-2* gene, which regulates developmental stages, and in turn, promotes virilization. The VBH-1: GFP localization technique shows that the fusion protein is co-located with P-particles, including proteins required for mature RNA and germline-specific proteins (PGL-1) [7].

Previous data suggest that the function of *laf-1* is required for embryonic development and sex differentiation in *C*. *elegans*, *laf-1* was identified in screens for dominant suppressors of the sterility produced by gain-of-function *fem-3* mutations and it is thought to promote male cell fates by negatively regulating expression of *tra-2* in both hermaphrodites and males [8]. It was expressed in somatic cells of the male gonads and hermaphroditic nematodes in all stages of development. In addition, Duan et al. investigated the expression patterns of *laf-1* gene from *Romanomermis wuchangensis* at different developmental stages by qRT-PCR, and detected the function of the sex differentiation gene using RNAi, but she did not show the specific function of the gene in *R*. *wuchangensis* [9]. Consequently, the function of *laf-1* gene in is not clear in mermithid nematode, we want to explore the function of *laf-1* gene in *O*. *sinensis*.

## Materials and methods

### *O*. *sinensis* and the host

*H*. *armigera* larvae, obtained from the Chinese Academy of Science (Wuhan, China), were used as hosts. A laboratory colony of *O*. *sinensis* was originally collected from Shangcai, Henan Province, China (114°54′E; 33°38′N), as described by Jiao et al. [10].

### Materials collection

Proteins from *O*. *sinensis* were frozen in liquid nitrogen and grinded in ice with an 800 μl grinding buffer (0.1 mol/ L NaCl, 0.01 mol/ L Tris (pH 7.0), 0.001 mol/ L EDTA (pH 8.0), and 100 μg/ ml PMSF), followed by sonication in ice with a certain amount of PMSF. Lysates were centrifuged at 12,000 rpm for 20 min at 4°C until the total protein was presented in the supernatant. The supernatant was boiled in an SDS sample buffer for 10 min at 100°C, then stored at -20°C until use.

A total RNA extraction kit (SV Total RNA Isolation System) and the pGEM-T Easy vector kit were purchased from Promega Corporation; a total RNA extraction kit (TIANGEN E.Z.NA. TM MicroElute Total RNA Kit, Beijing, China) was purchased from Omega Company; a Fluorescent Assay Kit (TIANGEN FastQuant RT Kit, Beijing, China), MLVs reverse transcriptase, ExTaqTM, pMDTM18-T vector connecting kit and gel extraction kit were purchased from Takara; SMARTTMRACE kit was purchased from Clontech Company. A Protein Marker was purchased form Fermentas. Pmal-C2x vector and Anti-MBP antibodies were purchased from NEB. DyLight 800 Goat anti-Mouse IgG (H + L) was purchased from KPL. DIG High Prime Labeling Kit I and Anti-Digoxigenin-AP Fab fragments were purchased from Roche. Primers were synthesized at Qingke Company.

### RNA extraction, cloning and sequence analysis of cDNA

Total RNA was extracted from each sample using Trizol reagents (Invitrogen, China), according to the manufacturer’s instructions, and then treated with DNase I (Invitrogen, China). First strand, complementary DNA was reverse-transcribed using the Maxime RT PreMix kit (TIANGEN, Beijing, China). The PCR products were resolved by electrophoresis on a 1% ethidium-bromide-stained agarose gel. Then, we performed reverse transcription reactions according to instructions. Purified DNA fragments were ligated into a pMD18-T vector and transformed into *Escherichia coli* DH5α competent cells. Single white colonies were randomly picked and cultured in 5 ml Luria–Bertani (LB) liquid medium containing ampicillin (100 mg/ml) and grown for 8 h at 37°C. The products were confirmed by sequencing at the GenScript biotechnology company (Nanjing, China). DNAman, Lasergen, and Clustalw biological software were used for sequencing analyses, using Blast search on the NCBI website (www.ncbi.nlm.nih.gov/BLAST). MEGA 6 was used to construct a phylogenetic tree using the neighbor-joining method, based on differentiation genes relative to other nematodes species. Domain prediction was performed using SMART (http://smart.embl-heidelberg.de).

### *Oslaf-1* full-length cDNA

The reverse transcription product with an oligo (dT) 24 primer and MLV reverse transcriptase (TaKaRa, Dalian, China) were used in PCR to amplify the target gene *Oslaf*-1 open reading frame according to the Takara (SYBR Premix Ex Taq II) manufacturer’s instructions. Based on the sequence of *Oslaf-1* found in our laboratory, the cDNA of *Oslaf-1* gene was obtained using a cDNA amplification kit (TaKaRa, Dalian, China). A pair of specific primers (Table 1) was designed to amplify the full-length cDNA using Primer Premier 5.0 software. The following reaction procedure was used for our PCR experiment for denaturing: 3 min at 95°C, 35 cycles for 30 s at 95°C, 30 s at 55°C, 30 s at 72°C, and 10 min at 72°C. The amplified product was cloned into a pMD18-T vector and transformed into the *E*. *coli* strain DH5α before sequencing.

**Table 1:** Information on primers.

**Primer name**		**Primer sequence**
RT-PCR	vS/vA	ATGGCNTGYGCNCARACNGG/ AARCCCATRTCYARCATNCGATC
5’RACE	5GSP/5NGSP	ACGTCGATCAATCGCCCAGGAGTA/ CAAAATGGCGGATTGTATGGGCGT
3’RACE	3GSP/3NGSP	ACCGAACGAAGTTGGAGGCAATGG/ CCGCCGTCGTCATTACCCGATAGC
specific	vL/ vR	CTATACCCACTGCATTAGAG/ CATTGCCTCCAACTTCGTTC
	aL/aR	ACACCGTTCCCATCTACGAA/ GTCCAAAGCGACATAGCACA
Probe	vA2/ vS2	CCCACAGTATCCTTGCTCC/ GACCCGTGAATTATCGTTG
Prokaryotic expression	*Oslaf-1*L/ *Oslaf-1*R	CGCGGATCCTTTTCCGATTTGAATATGCACCCTTG/CCCAAGCTTCTATACGTTCCCTACGCGACCGGTAC
RNAi	*Oslaf-1*L/ *Oslaf-1*R	TAATACGACTCACTATAGGGAGAGGAGCAAGGATACTGTGGG/TAATACGACTCACTATAGGGAGACCGCAGTGGCAACTAAAA

### The expression pattern of *Oslaf-1*

The quantitative real-time PCR (qRT-PCR) method was utilized to determine the expression pattern of *Oslaf-1* in sugarcane via SYBR Premix Ex Tap™ II (TaKaRa, Dalian, China). Each sample RNA from the embryos development, pre-parasitic juveniles (J2), parasitic juveniles (J3) (parasitic for two, four, and six days), post-parasitic juveniles (J4) (five and 15 days after prolapsing), adult females and males (five days after molting) phase, adult females and males five days after mating, gravid females were extracted from *O*. *sinensis* using E.Z.NA. TM MicroElute Total RNA Kit (Omega Company). Each test was replicated three times. We used vL/vR, and aL/aR primers to amplify the *Oslaf-1* gene (Table 1) and to amplify *actin*, which was used as an internal control, to quantify the relative transcript level of *Oslaf-1*. Based on the cDNA sequence, specific primers of the *Oslaf-1* gene (Table 1) were also designed. Reverse transcription was performed for qRT-PCR. The qRT-PCR was initiated with an activation step at 95°C for 2 min, followed by 40 cycles of 10 s at 95°C, 8 s at the Tm specific for the primer pairs used. A melting curve cycle was given at 95°C for 8 s, 58°C for 12 s with acquisitions 0.5 per°C from 95 to 58°C to confirm the amplification of a single product. The relative level of gene expression was calculated using the 2^−ΔΔCt^ formula of Livak and Schmittgen [11], calculated from three samples from independent experiments. Data were subjected to one-way analysis of variance followed by Tukey’s test for multiple comparisons, where differences were considered significant at P < 0.05.

### The expression of *Oslaf-1* in different tissues of *O*. *sinensis*

Tissue distributions of *Oslaf-1* mRNA were investigated in *O*. *sinensis*, using the semi-quantitative RT-PCR and using *β-actin* as a control. Each sample RNA were extracted from the testis, ovary, body wall, head, and tail. Each test replicated three times. Semi-quantitative RT-PCR was conducted as follows: 94°C for 3 min, 32 cycles of 94°C for 30 s, 58°C for 30 s, and 72°C for 30 s, ending with 10 min of extension at 72°C. PCR products were isolated with 1% agarose gel.

### The distribution of *Oslaf-1*

Immune serum was collected from immunized mice with the assistance of the Virus Research Institute in Wuhan and the purified protein was used to immunize BALB/c mice. Antibody was tested using the Western blot analysis. Anti-MBP-OsLAF-1 antiserum was obtained two months later, Western blot was performed according to standard protocols as previously described by Wei et al. [12].

### The localization of *Oslaf-1*

In situ hybridization on paraffin sections of embryos and on the embryos of medaka were carried out according to protocols described by Christine et al. [13]. Sense and antisense digoxigenin (DIG)-labeled RNA probes were synthesized with the DIG RNA labeling kit (Roche Diagnostics GmbH, Mannheim, Germany), following the manufacturer's instructions. The expression of the *Oslaf-1* transcript gene was determined according to Thompson et al. [14].

### Nematode soaking, FITC treatments and RNAi assay

*Oslaf-1* was determined using an RNAi experiment to determine the physiological function of sex differentiation. Double-stranded RNA (corresponding to *Oslaf-1*) was used in the soaking experiments, as described by Duan et al. [9].

### Statistical analysis

Using SPSS (SPSS Inc., Chicago, Illinois, U.S.A), significant differences between treated groups and control group were evaluated by Student's t-test at P < 0.05 and P < 0.01.

## Results

### Phylogenetic and sequence analysis of the *Oslaf-1*

**Fig 1:**
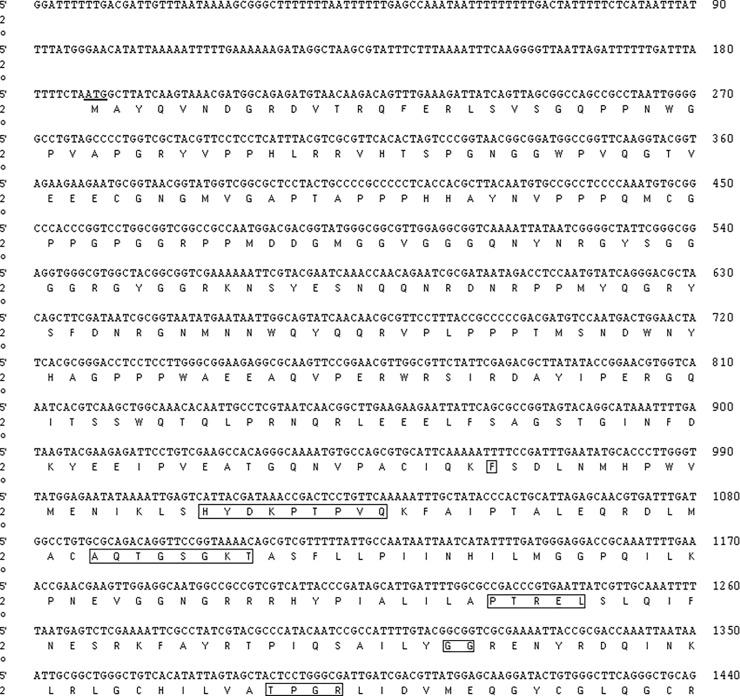
Nucleotide and deduced amino acid sequences of *Oslaf-1*. Nucleotides and amino acids are numbered along the right margin; the start codon, stop codon, and the plus tail signal ATTAAA are underlined; the DEAD-box domains are contained within the rectangles.

The full length of *Oslaf-1* cDNA is 2,596 bp with an open reading frame (ORF) that spans 2,235 bp. The ORF codes for a protein with 744 amino acid residues and a molecular weight of 82.5 kDa (Fig 1). The *Oslaf-1* contains all structural domains of the DEAD-box gene family: F,MYDKPTVG,AQTGSGKT,PTREL,GG,TPGR,DEAD,SAT,RGLD,HRIGRTGR. Therefore, it can be shown that the *Oslaf-1* gene belongs to the DEAD-box gene family. The motif (AQTGSGKT) corresponds with the motif (AXXXXGKT). It is located in the N-terminal (301–308) of *Oslaf-1* and is an ATPase A motif and an ATP binding site. The conserved region, located in the N-terminal (423–426) of *Oslaf-1*, is an ATPase B motif, which is the ATP hydrolysis site, a region widely found in all DEAD-box protein families. In some DEAD-box family proteins, the conserved regions (SAT and HRIGRTGR) are thought to be related to RNA binding and helicasing [15, 16].

**Fig 2:**
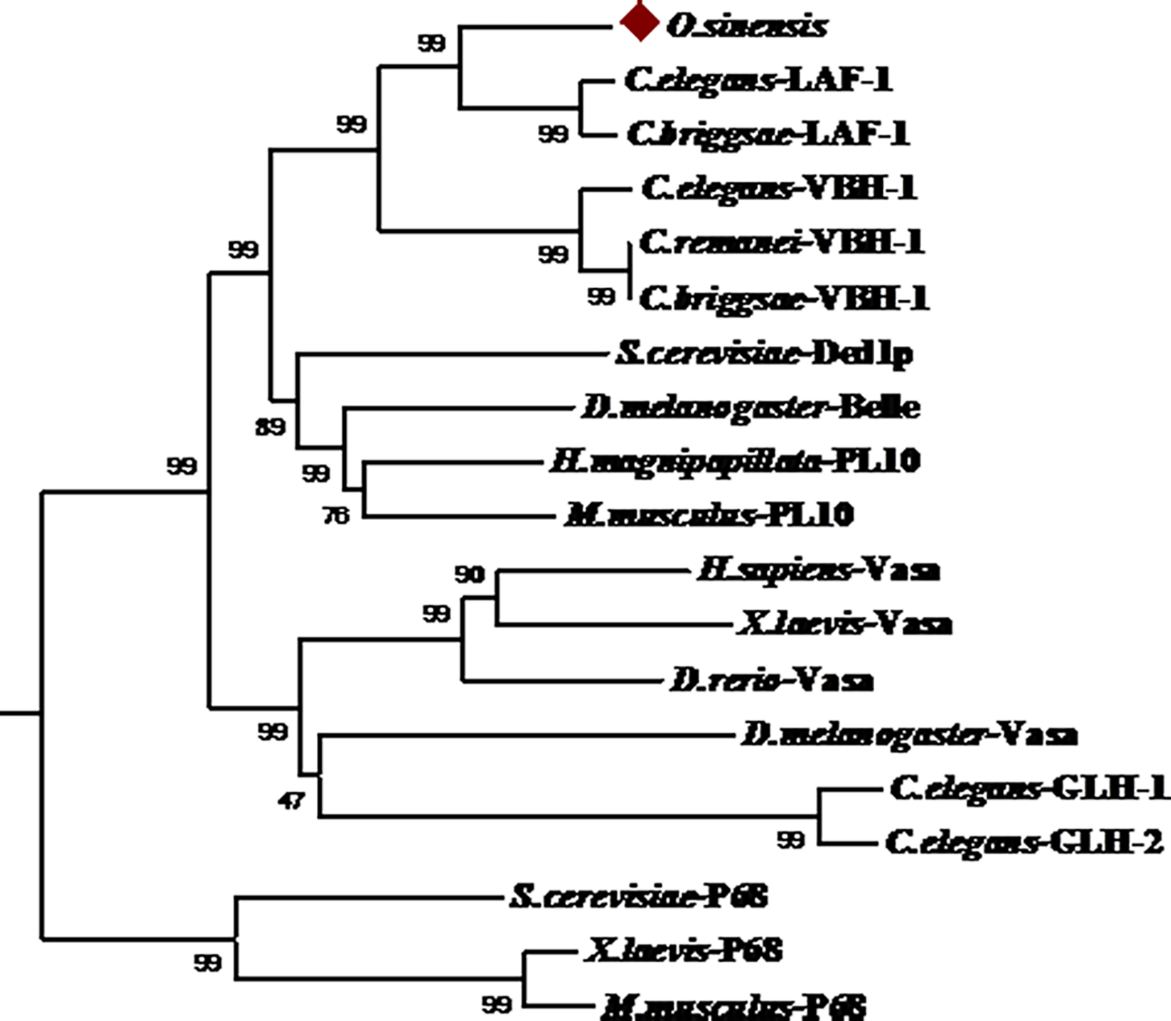
Phylogenetic analysis of the DEAD-box proteins, based on the conserved sequences of amino acids. **Sequence source in the evolutionary tree (GenBank Accession No. of proteins are).** *C*. *elegans*: LAF-1 (ACO56244), *C*. *briggsae*: LAF-1 (XP_0026395469), *C*. *elegans*: VBH-1 (NP_491112), *C*. *remanei*: VBH-1 (BP: CBP30447), *C*. *briggsae*: VBH-1 (XP_001668269), *Saccharomyces cerevisiae*: Ded1p (CAY86489), *Drosophila melanogaster*: Belle (NP_536783.1), *Hydra magnipapillata*: PL10 (BAB13306), *Mus musculus*: PL10 (NP_149068), *Homo sapiens*: Vasa (AAF86585), *Xenopus laevis*: Vasa (NP_001081728), *Danio rerio*: Vasa (NP_571132.1), *D*. *melanogaster*: Vasa (NP_723899.1), *C*. *elegans*: GLH-1 (NP_491963.1), *C*. *elegans*: GLH-2 (AAB03510), *S*. *cerevisiae*: P68 (NP_014287), *X*. *laevis*: P68 (NP_001081728), *M*. *musculus*: P68 (CAA46581.1). Branch lengths are proportional to the number of amino acid substitutions and the numbers on the branches represent percent bootstrap values.

We conducted phylogenetic and molecular evolutionary analyses of selected homologs of 4E-T from diverse animals using the software MEGA version 3.1 [17]. We selected some typical members of the DEAD-box family of proteins, such as Vasa, PL10, and three P68 subfamilies. When a phylogenic tree of the conserved sequences of amino acids was constructed using the neighbor-joining method, all Vasa, PL10, and P68 subfamily members clustered into a branch (Fig 2). Although they all belong to the DEAD-box family, there is a greater similarity between some members of the same subfamily than among members of other subfamilies. In addition, the *laf-1* gene shared high homology with the *VBH-1* gene of *C*. *elegans*, *C*. *remanei*, and *C*. *briggsae*, further indicating that the protein in our experiment belongs to the PL10 subfamily. Therefore, we determined that the gene was *Oslaf-1*.

**Fig 3:**
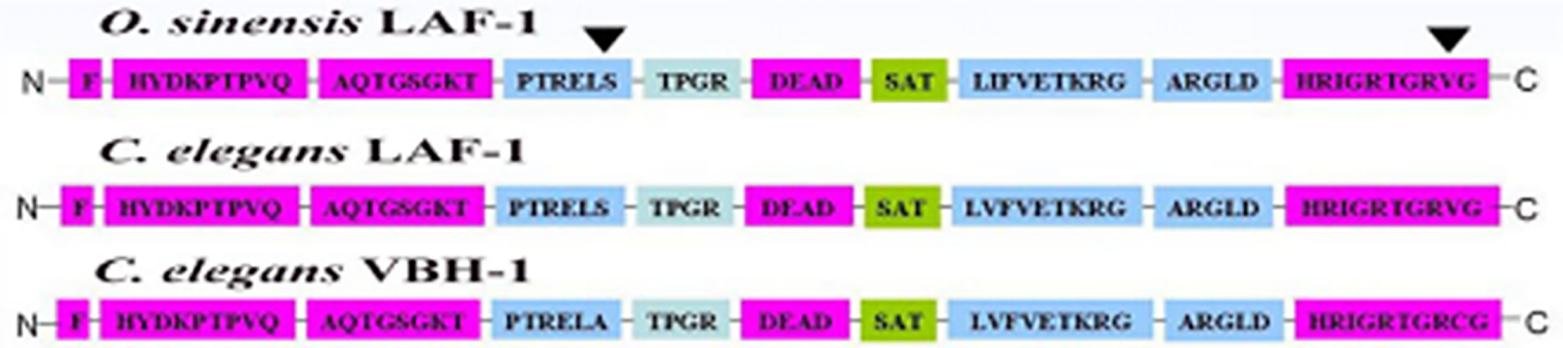
The comparison between the *Oslaf-1* of *O*. *sinensis* and the PL10 sub-family of *C*. *elegans* in conserved motifs of the protein.

The phylogenetic tree shows that DEAD-box family amino acid sequences were relatively conserved (Fig 2). The *Oslaf-1* gene from *O*. *sinensis* has the closest amino acid sequence identity (73%) to the sequence of the *laf-1* gene from *C*. *elegans*, followed by 66%, 64%, and 56% similarity to the sequences of *VBH-1* (*C*. *elegans*), *PL10* (*M*. *musculus*), and *Ded1p* (*S*. *cerevisiae*), respectively. Conserved motifs of the protein were compared among the *Oslaf-1* gene of *O*. *sinensis* and the *VBH-1、laf-1* of *C*. *elegans* (Fig 3). There is a high homology in the conserved motifs of the three proteins. This provides evidence that the gene cloned in *O*. *sinensis* is *Oslaf-1*.

### Expression patterns of the *Oslaf-1*

**Fig 4:**
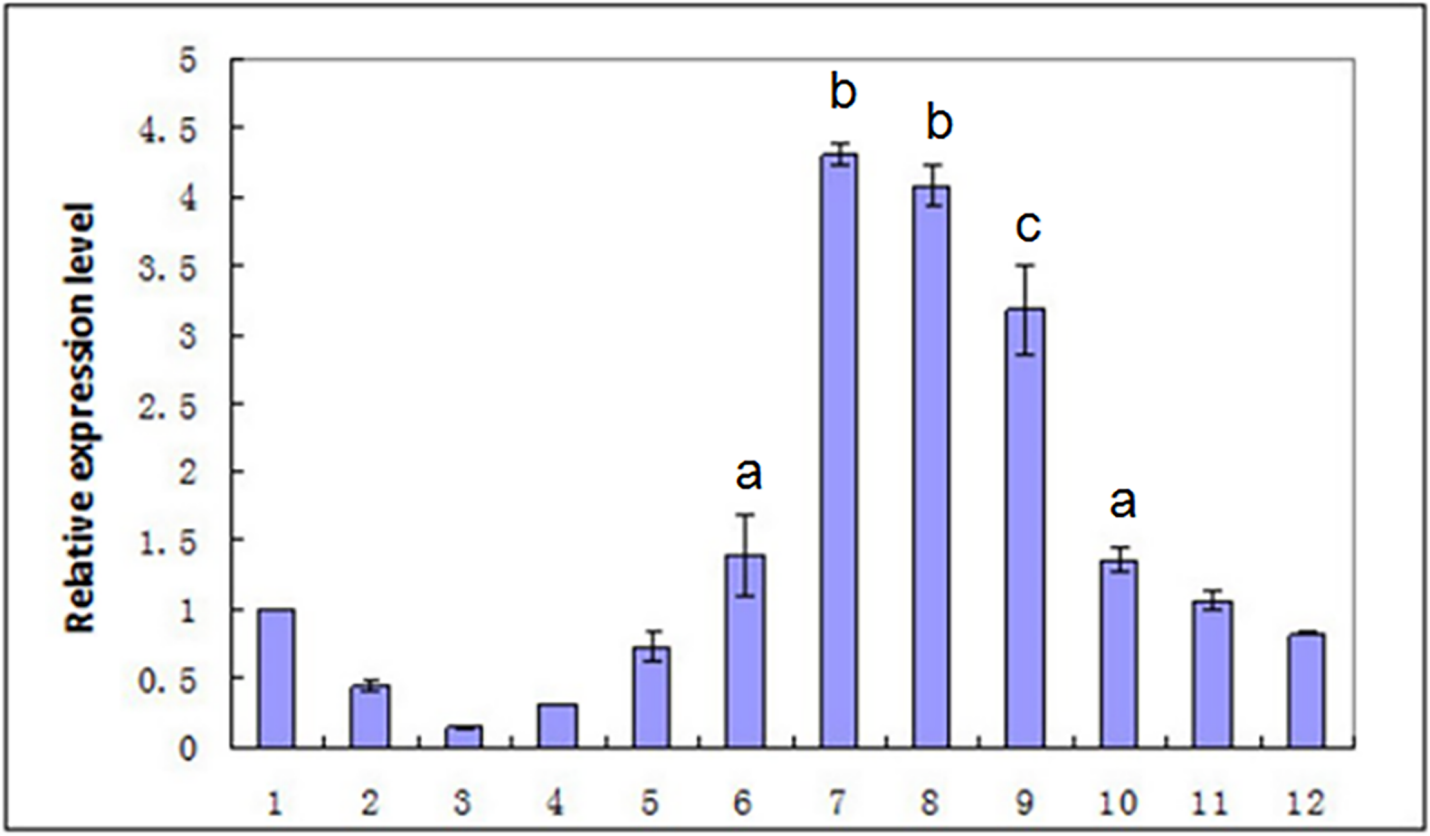
Analysis of the *Oslaf-1* expression at different developmental stages. 1: embryo development phase; 2: pre-parasitic juveniles (J2); 3–5: parasitic juveniles (J3), parasitic for two, four, and six days; 6–7: post-parasitic juveniles (J4) (five and 15 days after prolapsing); 8–9: adult females and males, five days after molting; 10–11: adult females and males five days after mating; 12: gravid females.

The qRT-PCR method was used to measure mRNA expression pattern of the *Oslaf-1* gene in *O*. *sinensis* (Fig 4). The qRT-PCR results revealed that the earliest period that the *Oslaf-1* transcript can be detected is during the embryo development phase, but there was no significant difference in gene expression level during the embryo development phase, pre-parasitic juveniles (J2), parasitic juveniles (J3), adult males and gravid females. The highest relative level of expression was during the post-parasitic stage (J4) (especially at 15 days after prolapsing) (P < 0.01). Following that, the degree of expression gradually decreased. However, expression level was significantly higher in females than in males (P < 0.05).

### Expression profiles of *Oslaf-1* for various tissues

**Fig 5:**
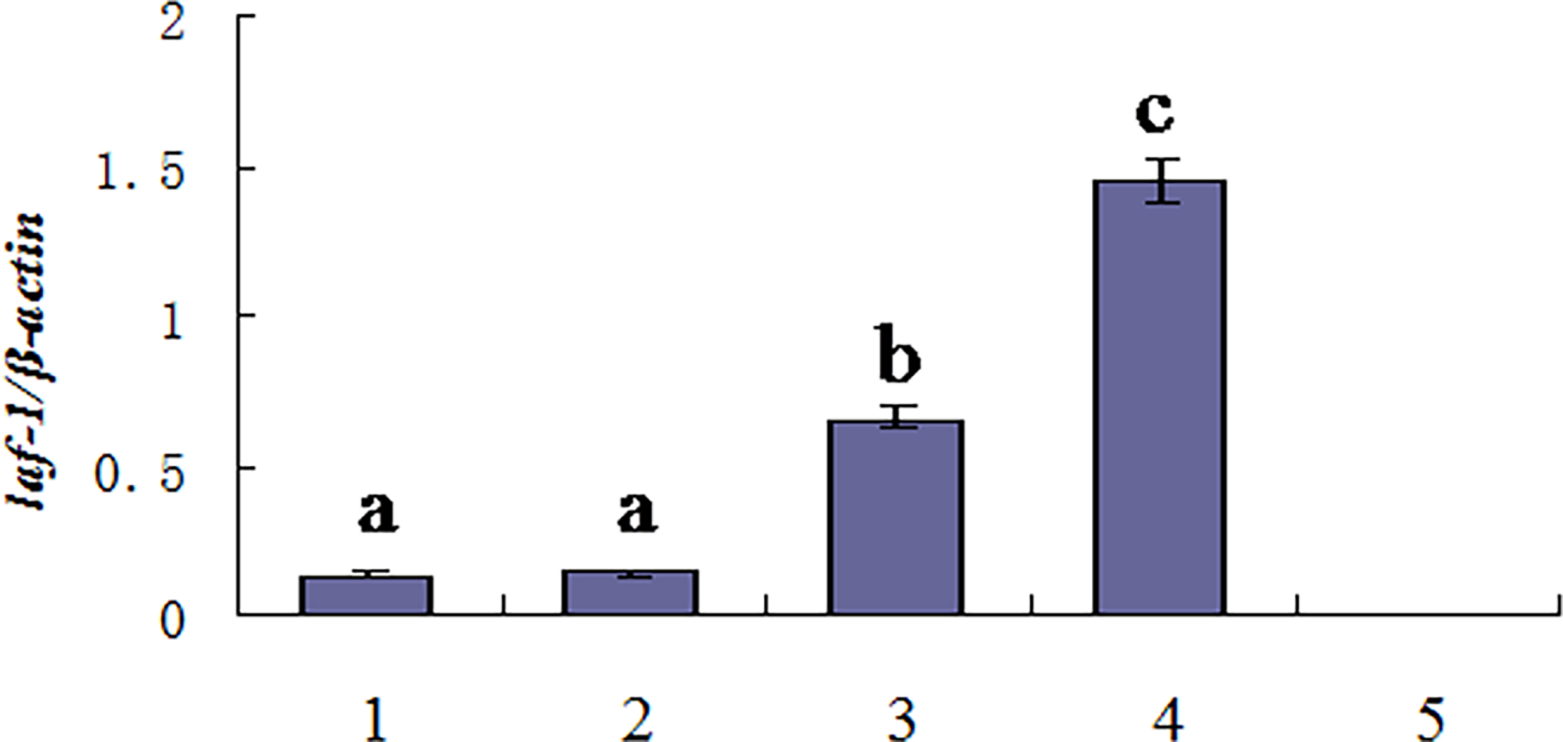
The expression of *Oslaf-1* in various tissues of *O*. *sinensis*. 1: head; 2: tail; 3: testis; 4: ovary; 5: integument.

The qRT-PCR revealed that *Oslaf-1* was expressed in all tissues we examined, including head, tail, testis, ovary, and integument. The gene was expressed strongest in both the testis and ovary (Fig 5), expressed relatively weakly in the head and tail, and relatively-weakly or not expressed in the integument. The relative expression of *Oslaf-1* in the ovary and testis was significantly higher than in other tissues (P < 0.05), and the signal in the ovary (1.4437 ± 0.0898) was significantly stronger than in the testis (0.6563 ± 0.0712) (P < 0.05). This result suggests that the gene is related to the functioning of the gonads, although the *Oslaf-1* transcript was not specifically expressed in gonads.

### Expression of recombinant *Oslaf-1* and Western blot analysis

#### Prokaryotic expression of the OsLAF-1 protein

**Fig 6:**
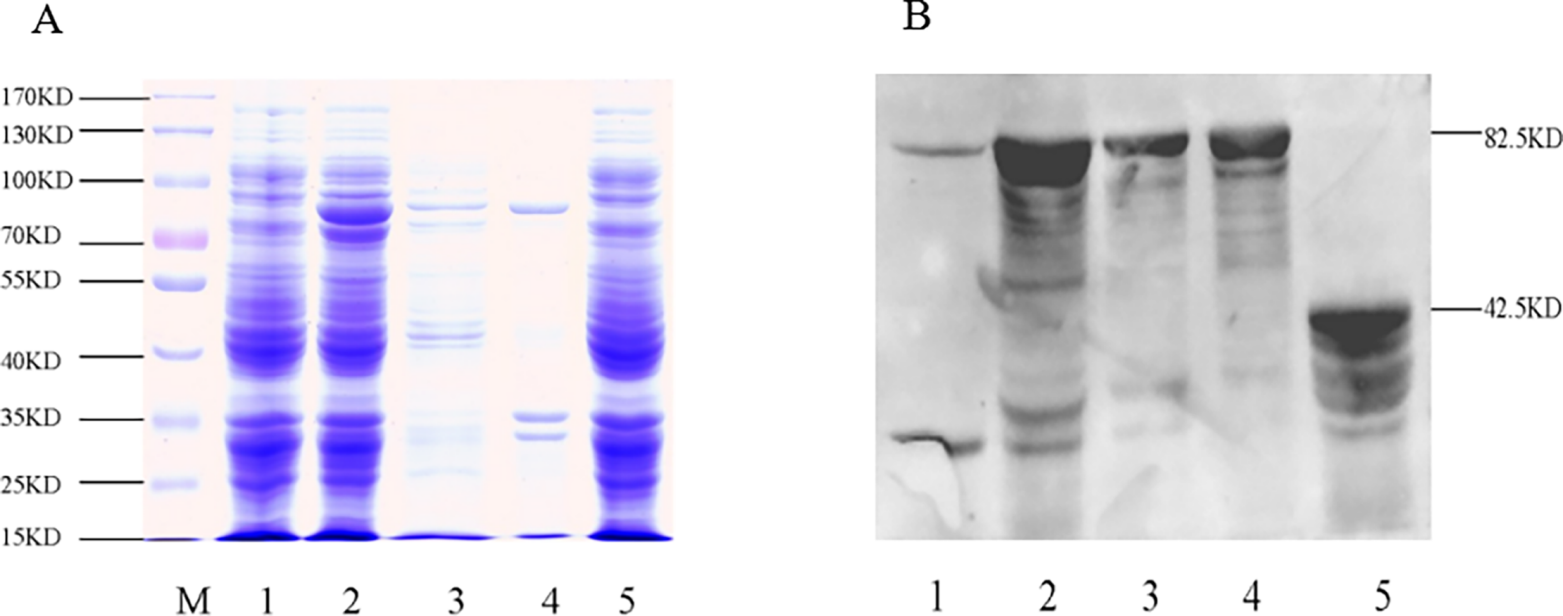
Expression of MBP fusion proteins and their assays with Western blot. Columns: (A) Expression of MBP-OsLAF-1 analyzed by 10% SDS–PAGE and (B) Identification by Western blot using anti-MBP monoclonal antibody; For both MBP and assays: M: high molecular weight marker; Lane 1: whole lysate of cells harboring PMAL-C2x-OsLAF-1 without IPTG treatment during induction; Lane 2: whole lysate of cells harboring PMAL-C2x-OsLAF-1 after induction with IPTG; Lane 3: lysate of soluble fraction of cells harboring PMAL-C2x-OsLAF-1 after induction with IPTG; Lane 4: lysates of insoluble fraction of cells harboring PMAL-C2x-OsLAF-1 after induction with IPTG; Lane 5: whole lysate of cells harboring PMAL-C2x after IPTG induction.

The MBP-OsLAF-1 fusion protein was obtained using PCR and cloned into the bacterial expression vector pET-32a. Recombinant plasmids were transformed into competent *E*. *coli* BL21 (DE3) cells and protein expression was induced by IPTG. Whole cell lysate was analyzed by SDS–PAGE and stained with Coomassie brilliant blue, which revealed a distinct band with a molecular mass of 82.5 kDa (in accordance with the predicted molecular mass). Cells of *E*. *coli*, BL21 transformed with pMAL-c2x vector (and induced with IPTG) produced a protein of 42.5 kDa (Fig 6A).

Recombinant protein (analyzed by Western blot) showed predicted bands of 82.5 kDa recombinant MBP-OsLAF-1 protein (Lane 2) and 42.5 kDa MBP protein (Lane 5) (Fig 6B). The dilution of anti-MBP and fluorescent-mouse secondary antibody was 1:3000 and 1:5000, respectively.

#### The preparation of the *Oslaf-1* antiserum and Western blot analysis of the specificity of antisera

**Fig 7:**
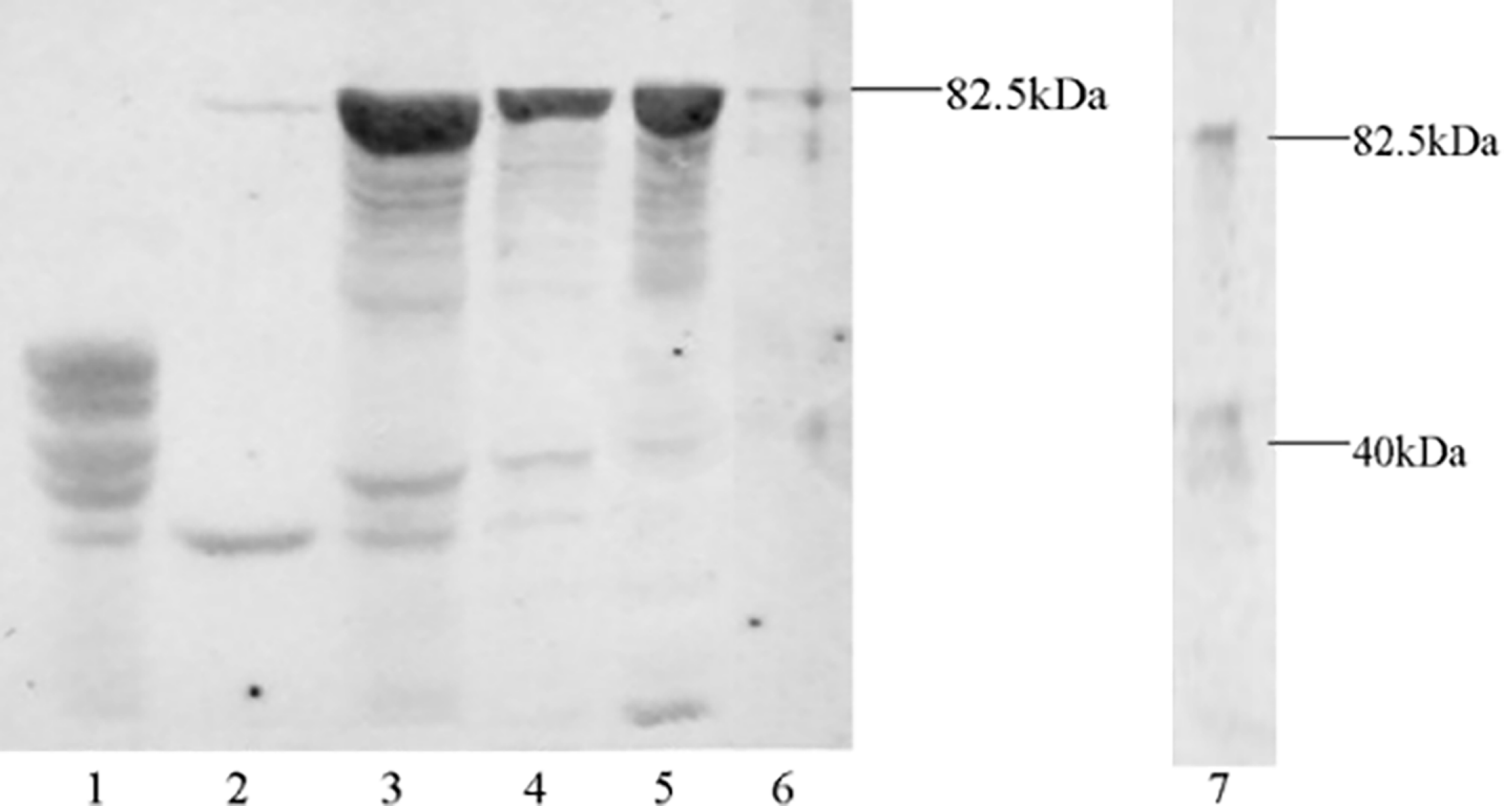
Western blot analysis of the specificity of antisera. Columns: 1: whole lysate of cells harboring PMAL-C2x after IPTG induction; 2: whole lysate of cells harboring PMAL-C2x-OsLAF-1 without IPTG treatment during induction; 3: whole lysate of cells harboring PMAL-C2x-OsLAF-1 after induction with IPTG; 4: lysate of soluble fraction of cells harboring PMAL-C2x-OsLAF-1 after induction with IPTG; 5: lysate of insoluble fraction of cells harboring PMAL-C2x-OsLAF-1 after induction with IPTG; 6: purified protein eluted from amylose column with maltose, purification of the PMAL-C2x-OsLAF-1; 7: purified protein after Factor Xa cleavage.

Our antibody tests showed that the MBP-OsLAF-1 could be recognized by the polyclonal antibody against the fusion protein (Fig 7). However, no obvious band was detected in the groups of lysates we tested from bacteria without induction or from those only expressing the MBP protein. These results indicated that the antiserum had high specificity for the *Oslaf-1* gene. The dilution of the polyclonal antibody and fluorescent-mouse secondary antibody was 1:3000 and 1:5000, respectively.

### The distribution of OsLAF-1 protein in different tissues of *O*. *sinensis*

**Fig 8:**
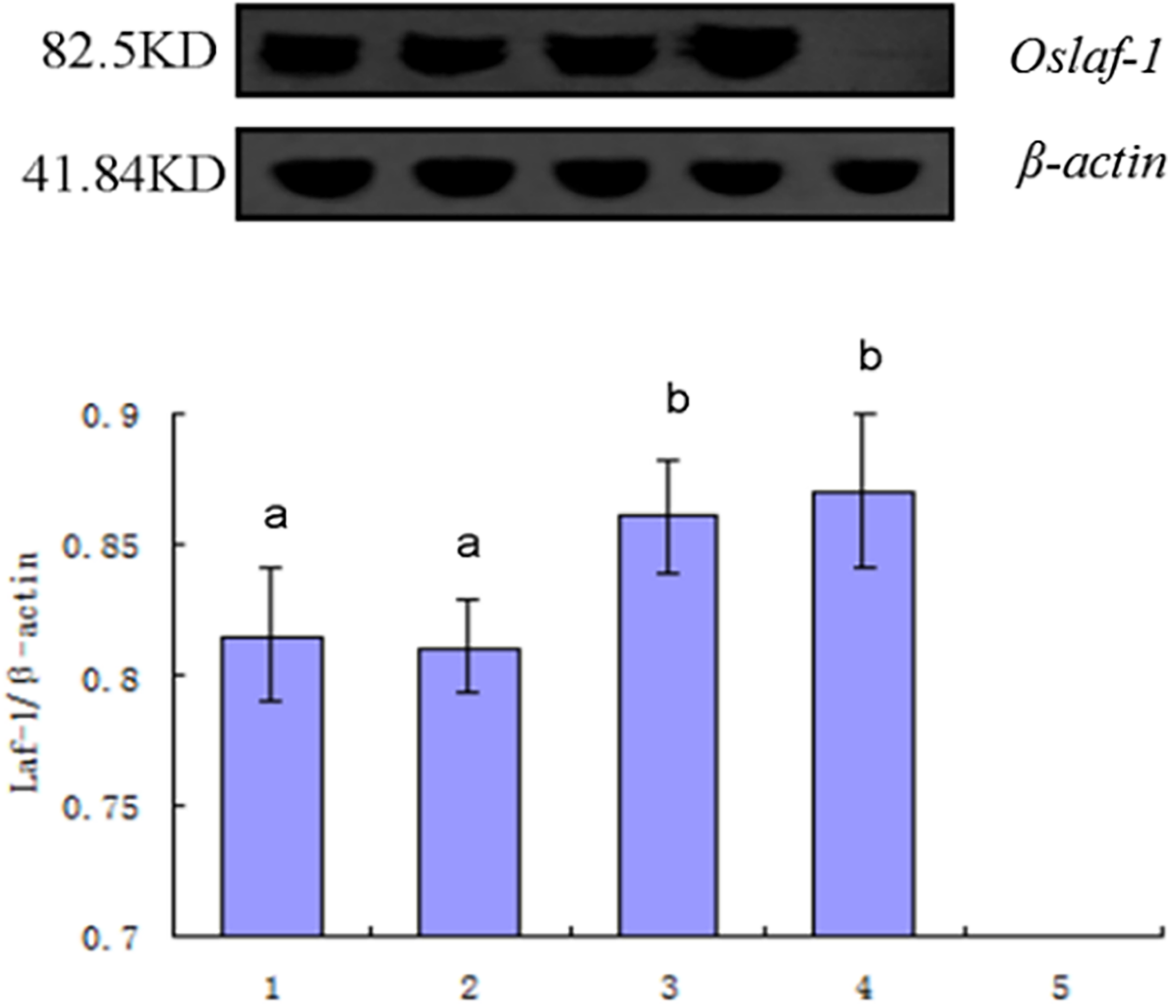
The distribution of OsLAF-1 in various parts of *O*. *sinensis*. Columns: 1: head; 2: tail; 3: testis; 4: ovary; 5: integument.

The results of the Western blot test showed that the *Oslaf-1* gene was expressed in the testis, ovary, head, and tail, whereas there was low or no expression in the integument (Fig 8). The signal in the testis and ovary was significantly higher than in the head and tail (P < 0.05), these results were consistent with previous studies in qRT-PCR, indicating that the gene is related with the functioning of the gonads.

### In situ hybridization of embryos

**Fig 9:**
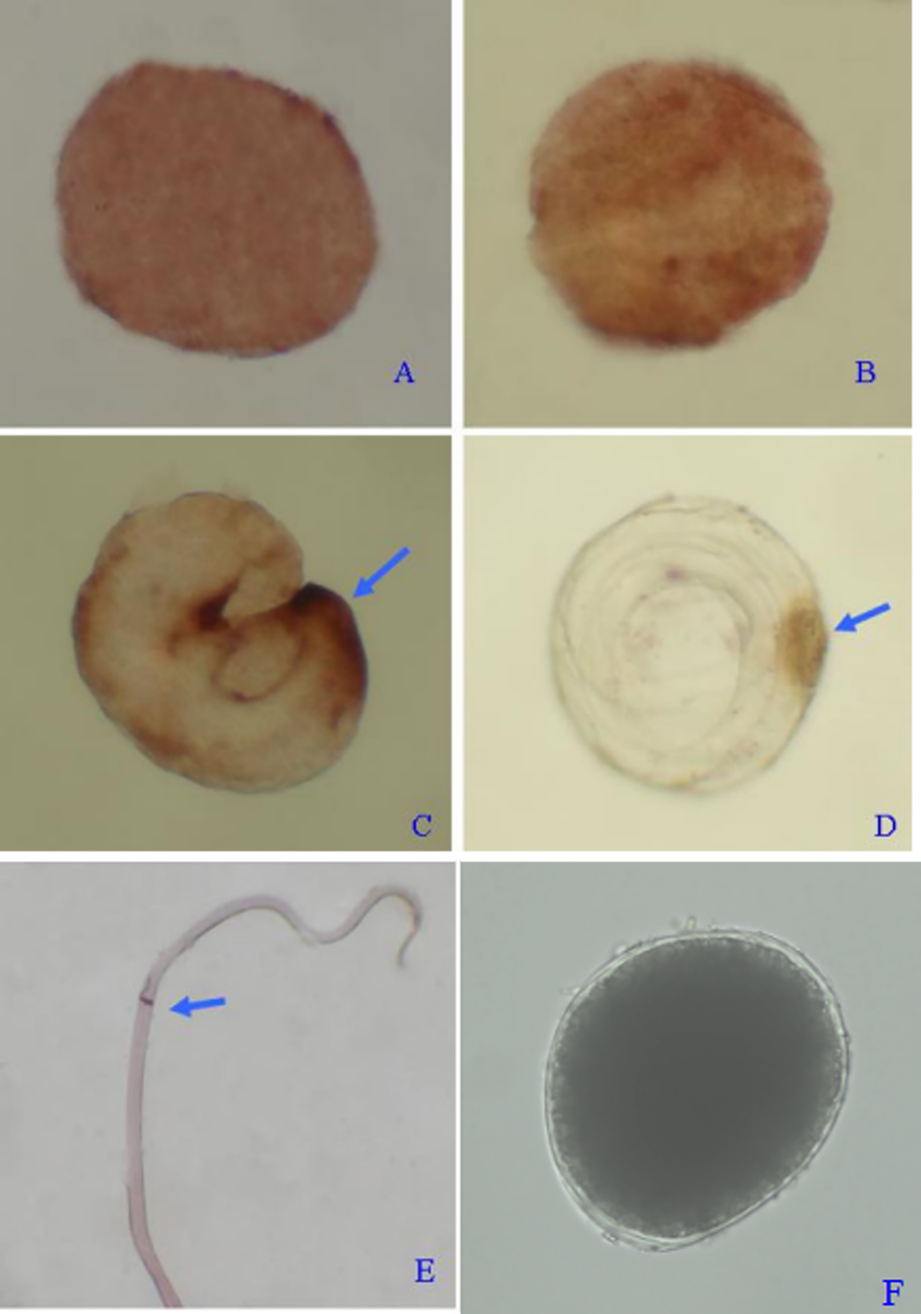
The ISH result of embryos of *O*. *sinensis* in different stages. A: unicellular stage; B: blastula stage; C: gastrula stage; D: juvenile I stage; E: pre-parasitic juvenile stage; F: control (blastula stage). Note: Picture A-D, F (40 × magnification), E (1 × magnification).

The probes labeled by DIG were used for hybridization with embryos and pre-parasitic juveniles. NBT/BCIP staining showed positive signals (Fig 9). The results showed that the red-brown positive signals could be detected in all stages of embryonic development (Fig 9). In the early stages of embryogenesis, the signals were distributed widely in the embryonic blastomeres (Fig 9A and 9B). As embryogenesis progressed, signals gathered in the certain parts of the embryos, which may be in gonads (Fig 9C). In pre-parasitic juveniles, the signals mainly concentrated in the reproductive rudiment (Fig 9D and 9E). Therefore, we hypothesize that the expression of the *Oslaf-1* transcript is closely related with early embryogenesis and gonadal development.

### RNAi of *Oslaf-1* of *O*. *sinensis*

**Fig 10:**
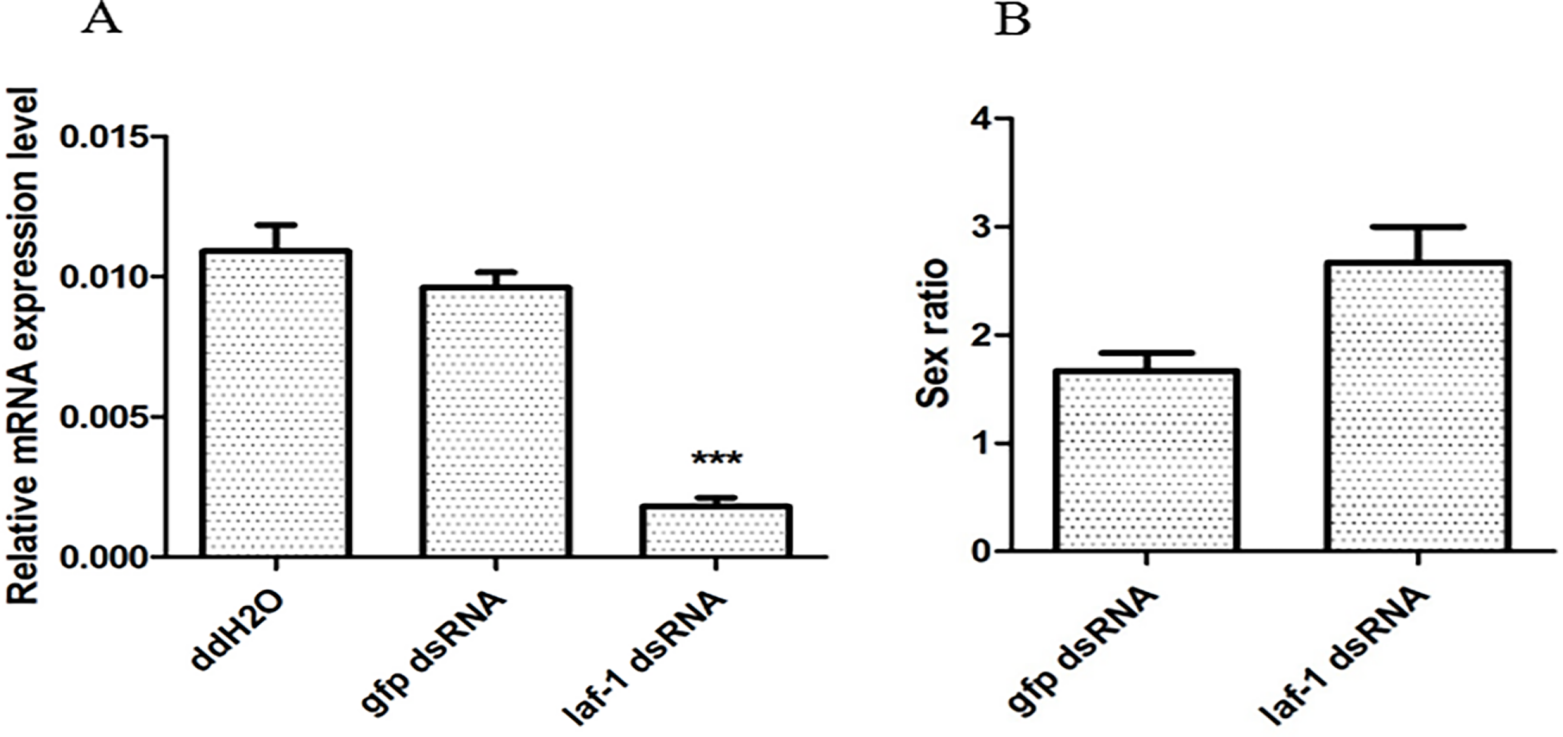
Sex ratio displayed (A) and relative mRNA expression level (B) by *O*. *sinensis* following soaking for 16 h in dsRNA of *Oslaf-1* (*P < 0.05, **P < 0.01, ***P < 0.001).

RNAi results revealed that the sex ratio of *O*. *sinensis* (determined from RNAi treated groups soaked in dsRNA of *Oslaf-1*) demonstrated a slight, there was no significant difference compared with the control group (Fig 10A). In order to detect whether RNAi successfully silenced the gene, we performed fluorescence quantitative experiments on the interfering nematodes. The results compared with the control group showed a significant difference and the knockdown effect is obvious (Fig 10B).

## Discussion

Helicases are enzymes that can separate duplex oligonucleotides in a NTP-dependent fashion and are essential in all aspects of DNA and RNA metabolism [18]. This protein contains two categories: DNA helicase and RNA helicase [19]. The classification of these proteins into three superfamilies and two families (herein named SF1–SF5) is based on proteins biological occurrence and characteristics of conserved motifs in the primary sequence [20]. According to the classification of Gorbalenya and Koonin, DEAD-box and related DEAH, DExH, and DExD families are members of SF2, sharing eight conserved motifs [21, 22]. The DEAD-box family is the largest family by far, and is characterized by the presence of nine conserved motifs that have been shown to be involved in ATPase and helicase activities and in their regulation [23]. Some of the conserved motifs also participate in RNA binding.

The DEAD-box proteins form a widely-dispersed family of proteins found in all eukaryotes, and most prokaryotes [24, 25]. The DEAD-box proteins were first identified as a distinct family in the late 1980s when alignments were discovered, based on eight homologues of the yeast eIF4A translation initiation factor [6]. The name of the family was derived from the amino-acid sequence D-E-A-D (Asp–Glu–Ala–Asp) of its Walker B motif.

The DEAD-box proteins participate in nearly all aspects of RNA metabolism [22], such as RNA transcription regulation, mRNA precursor splicing, transporting and maturation, ribosome biogenesis and assembly, initiation of translation of the protein, RNA degradation, and the expression of organelle genes [17, 26]. In addition, the proteins also play an important role in cell growth and differentiation. Despite the fact that DEAD-box proteins are involved in many diverse cell mechanisms, their precise roles, functions, and regulation are largely unknown [22].

Phylogenetic analyses show that the DEAD-box helicase family of proteins are generally divided into Vasa, PL10, P68, and the eIF4A subfamily. In our experiment, the *Oslaf-1* gene from *O*. *sinensis* belongs to the PL10 subfamily. It is very similar to the protein LAF-1 and VBH-1 in *C*. *elegans*, PL10 in mouse, and Ded1p in yeast, with 73%, 66%, 64%, and 56% similarity, respectively.

In qRT-PCR experiments, the gene expression of *O*. *sinensis* in the post-parasitic period and ovarian were the highest. We found that the *Oslaf-1* gene was highly expressed in the testis and ovary, but that expression was relatively weak in the head and tail (P < 0.05), whereas there was low or no expression in the integument (by qRT-PCR and Western blot). Our result was similar to the gene expression of PL10 subfamily of the DEAD-box family. The *PL10* gene in mice is only expressed in male germ cells in the early stage of embryogenesis, and functions in transcription regulation during spermatogenesis [27]. Some genes, such as *An3* in *Xenopus laevis*, *PL10a* in *zebrafish*, and *PL10* in *Rana temporaria* are expressed in the testis, ovary, brain, heart, intestines, and other tissues of adults [28, 29]. In addition, the gene *laf-1* in *C*. *elegans* is expressed in eggs, L1–L4 in larvae, young adults, in the gonads of males, and in somatic cells of hermaphrodites [30].

Salinas et al. find that VBH-1 protein is specially expressed in the gonadal of *C*. *elegans* and is also detected in the oogenesis and spermatogenesis [31]. However, immunofluorescence show that it can express in the primary spermatocyte and secondary spermatocyte but not in mature sperm, which indicate that VBH-1 protein may associate with occurrence and development of germ cells of *C*. *elegans*, and play an important role in reproductive development as Cre-VBH-1 proteins in *C*. *remanei* [27]. Immunolocalization reveal that VBH-1 and Cre-VBH-1 proteins are weakly expressed in somatic cells, suggesting that these two proteins may also function in tissues outside nematode’s germline [32].

The results of Western blot showed that *Oslaf-1* expressed in head, tail, testis and ovary of *O*. *sinensis* but no in its wall, and it expressed significantly higher in gonad than head and tail (P < 0.05) which was similar to the RT-PCR results. The results found in *O*. *sinensis* are consistent with the conclusions that *VBH-1* and *Cre-VBH-1* are function in gonad development of *C*. *elegans* and *C*. *remanei* respectively [27, 32, 33]. Genetic analysis shows that LAF-1 protein can promote *C*. *elegans* masculinization and it plays an important role in genital sex determination and embryo development in *C*. *elegans* [30]. Beside, *laf-1* gene is also expressed outside of the gonads in *C*. *elegans*. In this study, *Oslaf-1* gene expressed higher in gonads and it also detected in other tissues in *O*. *sinensis*. All of this are same with the finding in previous reports [33, 34], we speculate that OsLAF-1 protein is closely related to gonadal function in *O*. *sinensis*.

In situ hybridization showed that the *Oslaf-1* gene transcripts were not widely distributed in the gastrula stage, especially in juvenile Stage I, where the signal was focused on the reproductive rudiment. This indicates that the *Oslaf-1* gene is related to the development of gonads, which is consistent with the function of *VBH-1* and *laf-1* genes in the gonads of *C*. *elegans* [30, 31]. These gene transcripts were widely distributed in blastomeres (unicellular and four cellular stages), and in later stages, gradually concentrating in the reproductive rudiment. Furthermore, we found that the gene was a maternal gene that participates in the process of regulating the early embryogenesis and gonadal development of *O*. *sinensis*.

*O*. *sinensis* is an insect pathogen nematode whose sex determination occurs during parasitism, depending on the nutrient competition pressure (environmental determinism) of parasitic age. The growth and development of genital organs in *O*. *sinensis* occurred after they emerge from the host rather than the parasitism stage [35]. Our study (Figs 4–9) revealed that *laf-1*, a maternal gene, was associate with the development of gonadal for that was highly expressed in the ovary (P < 0.05). Fig 4 (3–5) shows that there was no significant differences in *laf-1* expression at parasitic stage, suggesting that was not play a direct role in sex-determining process of *O*. *sinensis*, and the RNAi experiment give the same conclusion.

## Conclusions

In order to study the mechanism of gender differentiation and gonadal development, we explored the *laf-1* gene, and the result shows that *laf-1* plays an important role in the development of gonad in the nematode but not associated with sex determination, and other functions of *laf-1* need to be further studied.

## References

[1] Wang G, Chen Q, Chen G. In vitro cultivation of the entomogenous nematode *Ovomermis sinensis*. Acta Zoologica Sinica. 2001; 47: 235–239.

[2] Platzer EG. Mermithid nematodes. Journal of the American Mosquito Control Association. 2007; 23:58–64. doi: 10.2987/8756-971X(2007)23[58:MN]2.0.CO;2 17853598 10.2987/8756-971X(2007)23[58:MN]2.0.CO;2

[3] Petersen JJ. Comparative susceptibility of larval mosquitoes exposed separately by instar or in mixed populations to the nematode *Romanomermis culicivorax*. Journal of Nematology. 1981; 13: 228–229. 19300751 PMC2618071

[4] Chaudhary BR, Singh HV. Nutritional control on sex differentiation in the Filamentous Green Alga Oedogonium hatei Kam. Archiv Für Protistenkunde Protozoen Algen Pilze. 1988; 136: 389–392.

[5] Stuart RJ, Hatab MA, Gaugler R. Sex ratio and the infection process in entomopathogenic nematodes: are males the colonizing Sex. Journal of Invertebrate Pathology. 1998; 72: 288 doi: 10.1006/jipa.1998.4789 9784353 10.1006/jipa.1998.4789

[6] Linder P, Lasko PF, Ashburner M, Leroy P, Nielsen PJ, Nishi K, et al Birth of the D-E-A-D box. Nature. 1989; 337: 121 doi: 10.1038/337121a0 2563148 10.1038/337121a0

[7] Marracci S, Casola C, Bucci S, Ragghianti M, Ogielska M, Mancino G, et al Differential expression of two *vasa/PL10*-related genes during gametogenesis in the special model system Rana. Development Genes & Evolution. 2007; 217: 395–402. 17333258 10.1007/s00427-007-0143-6

[8] Goodwin EB, Hofstra K, Hurney CA, Mango S, Kimble J. A genetic pathway for regulation of *tra-2* translation. Development. 1997; 124:749–758. 9043090 10.1242/dev.124.3.749

[9] Duan M, Xiong J, Lu D, Wang G, Hui A. Transcriptome sequencing analysis and functional identification of sex differentiation genes from the mosquito parasitic nematode, *Romanomermis wuchangensis*. PloS One. 2016; 11: e0163127 doi: 10.1371/journal.pone.0163127 27662191 10.1371/journal.pone.0163127 PMC5035087

[10] Jiao Z, Feng X, Tao S, Wang G. Nutrient-dependent patterns in the sex ratio of (Nematoda: Mermithidae), a biological control agent of (Lepidoptera: Noctuidae). Biocontrol Science and Technology. 2016; 26: 1–21.

[11] Livak KJ, Schmittgen TD. Analysis of relative gene expression data using real-time quantitative PCR and the 2(-Delta Delta C (T)) Method. Methods. 2012; 25: 402–408. 10.1006/meth.2001.1262 11846609

[12] Wei Y, Zhang Z, Liao H, Wu L, Wu X, Zhou D, et al Nuclear estrogen receptor-mediated Notch signaling and GPR30-mediated PI3K/AKT signaling in the regulation of endometrial cancer cell proliferation. Oncology Reports. 2012; 27: 504–510. doi: 10.3892/or.2011.1536 22075757 10.3892/or.2011.1536

[13] Thisse C, Thisse B. High-resolution in situ hybridization to whole-mount *zebrafish* embryos. Nature Protocols. 2008; 3: 59–69. doi: 10.1038/nprot.2007.514 18193022 10.1038/nprot.2007.514

[14] Thompson RC, Deo M, Turner DL. Analysis of microRNA expression by in situ hybridization with RNA oligonucleotide probes. Developmental Dynamics. 2006; 235: 2538–2548. doi: 10.1002/dvdy.20847 16736490 10.1002/dvdy.20847

[15] Pause A, Sonenberg N. Mutational analysis of a DEAD box RNA helicase: the mammalian translation initiation factor eIF-4A. Embo Journal. 1992; 11: 2643–2654. 1378397 10.1002/j.1460-2075.1992.tb05330.x PMC556740

[16] Pause A, Méthot N, Sonenberg N. The HRIGRXXR region of the DEAD box RNA helicase eukaryotic translation initiation factor 4A is required for RNA binding and ATP hydrolysis. Molecular & Cellular Biology. 1993; 13: 6789–6798. 8413273 10.1128/mcb.13.11.6789-6798.1993 PMC364741

[17] Rocak S, Linder P. DEAD-box proteins: the driving forces behind RNA metabolism. Nature Reviews Molecular Cell Biology. 2004; 5: 232–241. doi: 10.1038/nrm1335 14991003 10.1038/nrm1335

[18] Gustafson EA, Wessel GM. DEAD-box helicases: posttranslational regulation and function. Biochemical & Biophysical Research Communications. 2010; 395: 1–6. 20206133 10.1016/j.bbrc.2010.02.172 PMC2863303

[19] Tuteja N, Tarique M, Banu MSA, Ahmad M, Tuteja R. Pisum sativum p68 DEAD-box protein is ATP-dependent RNA helicase and unique bipolar DNA helicase. Plant Molecular Biology. 2014; 85: 639–651. doi: 10.1007/s11103-014-0209-6 24908423 10.1007/s11103-014-0209-6

[20] Gorbalenya AE, Koonin EV. Helicases: amino acid sequence comparisons and structure-function relationships. Current Opinion in Structural Biology. 1993; 3: 419–429.

[21] Tanner NK, Linder P. DExD/H box RNA helicases: from generic motors to specific dissociation functions. Molecular Cell. 2001; 8: 251 11545728 10.1016/s1097-2765(01)00329-x

[22] Cordin O, Banroques J, Tanner NK, Linder P. The DEAD-box protein family of RNA helicases. Gene. 2006; 367: 17 doi: 10.1016/j.gene.2005.10.019 16337753 10.1016/j.gene.2005.10.019

[23] Tanner NK, Cordin O, Banroques J, Doère M, Linder P. The Q Motif: A newly identified motif in DEAD box helicases may regulate ATP binding and hydrolysis. Molecular Cell. 2003; 11: 127–138. 12535527 10.1016/s1097-2765(03)00006-6

[24] Aubourg S, Kreis M, Lecharny A. The DEAD box RNA helicase family in *Arabidopsis thaliana*. Nucleic acids research. 1999; 27: 628 9862990 10.1093/nar/27.2.628 PMC148225

[25] Cruz JDL, Kressler D, Linder P. Unwinding RNA in *Saccharomyces cerevisiae*: DEAD-box proteins and related families. Trends in Biochemical Sciences. 1999; 24(5): 192–198. 10322435 10.1016/s0968-0004(99)01376-6

[26] Malygin AA, Parakhnevitch NM, Ivanov AV, Eperon IC, Karpova GG. Human ribosomal protein S13 regulates expression of its own gene at the splicing step by a feedback mechanism. Nucleic Acids Research. 2007; 35(19):6414–6423. doi: 10.1093/nar/gkm701 17881366 10.1093/nar/gkm701 PMC2095825

[27] Leroy P, Alzari P, Sassoon D, Wolgemuth D, Fellous M. The protein encoded by a murine male germ cell-specific transcript is a putative ATP-dependent RNA helicase. Cell. 1989; 57: 549 2720782 10.1016/0092-8674(89)90125-6

[28] Gururajan R, Perryo'Keefet H, Melton DA, Weeks DL. The *Xenopus* localized messenger RNA An3 may encodean ATP-dependent RNA helicase. Nature. 1991; 349: 717–719. doi: 10.1038/349717a0 1996140 10.1038/349717a0

[29] Olsen LC, Aasland R, Fjose A. A vasa-like gene in *zebrafish* identifies putative primordial germ cells. Mech Dev. 1997; 66: 95 9376327 10.1016/s0925-4773(97)00099-3

[30] Hubert A, Anderson P. The *C*. *elegans* sex determination gene *laf-1* encodes a putative DEAD-box RNA helicase. Developmental Biology. 2009; 330: 358–367. doi: 10.1016/j.ydbio.2009.04.003 19361491 10.1016/j.ydbio.2009.04.003 PMC2802855

[31] Salinas LS, Maldonado E, Macías-Silva M, Blackwell TK, Navarro RE. The DEAD box RNA helicase *VBH-1* is required for germ cell function in *C*. *elegans*. Genesis. 2007; 45: 533–546. doi: 10.1002/dvg.20323 17868112 10.1002/dvg.20323

[32] Salinas LS, Franco CA, Villanueva CE, Maldonado E, Navarro RE. Germ cell survival in *C*. *elegans* and *C*. *remanei* is affected when the DEAD box RNA helicases *VBH-1* or *Cre-VBH-1* are silenced. Genesis. 2012; 50(11): 801–818. doi: 10.1002/dvg.22043 22674898 10.1002/dvg.22043

[33] Zhao NN. The expression analysis of laf-1 of DEAD-box family from Ovomermis sinensis. M.Sc. Thesis. Central China Normal University. 2010; Available from: http://www.zhizhen.com/detail_38502727e7500f2669f33b81baeaeef2181d8c5183f11fd31921b0a3ea255101928fa69a765a3d2daa387d79f490a2074c8a1c8dd777f35abb0449af329be7a91fcc0cf7151ae782fd1c0138e2d1eacd.

[34] Wang WN. Cloning and expression of DEAD-box gene and β-actin gene from two species of mermithidae. M.Sc. Thesis. Central China Normal University. 2009; Available from: http://www.zhizhen.com/detail_38502727e7500f26a1282ac060f4289e78e2c9b7ecc817951921b0a3ea255101928fa69a765a3d2de7a6c4baef8a40a1b4ebbe39c4013efa1d7f07be5b891f602eae9643f923623f487122744cb13d18?&apistrclassfy=0_15_12.

[35] Li JL, Wang GX, Wang W, Liu XS, Qiao ZX, Zhao S. Changes of the morphological and chemical substances of the *Ovomermis sinensis* during gonadial development. Journal of Central China Normal University. 2006; 40(2):265–269.

